# Management of paediatric tibial fractures using two types of circular external fixator: Taylor spatial frame and Ilizarov circular fixator

**DOI:** 10.1007/s11832-014-0583-2

**Published:** 2014-04-19

**Authors:** Suhayl Tafazal, Sanjeev S. Madan, Farhan Ali, Manoj Padman, Simone Swift, Stanley Jones, James A. Fernandes

**Affiliations:** Department of Paediatric Orthopaedics, Sheffield Children’s Hospital, Western Bank, Sheffield, S10 2TH UK

**Keywords:** Tibial fracture, Ilizarov circular fixator, Taylor spatial frame

## Abstract

**Background:**

The use of circular fixators for the treatment of tibial fractures is well established in the literature. The aim of this study was to compare the Ilizarov circular fixator (ICF) with the Taylor spatial frame (TSF) in terms of treatment results in consecutive patients with tibial fractures that required operative management.

**Method:**

A retrospective analysis of patient records and radiographs was performed to obtain patient data, information on injury sustained, the operative technique used, time duration in frame, healing time and complications of treatment. The minimum follow-up was 24 months.

**Results:**

Ten patients were treated with ICF between 2000 and 2005, while 15 patients have been treated with TSF since 2005. Two of the 10 treated with ICF and 5 of the 15 treated with TSF were open fractures. All patients went on to achieve complete union. Mean duration in the frame was 12.7 weeks for ICF and 14.8 weeks for the TSF group. Two patients in the TSF group had delayed union and required additional procedures including adjustment of fixator and bone grafting. There was one malunion in the TSF group that required osteotomy and reapplication of frame. There were seven and nine pin-site infections in the ICF and TSF groups, respectively, all of which responded to antibiotics. There were no refractures in either group.

**Conclusion:**

In an appropriate patient, both types of circular fixator are equally effective but have different characteristics, with TSF allowing for postoperative deformity correction. Of concern are the two cases of delayed union in the TSF group, all in patients with high-energy injuries. We feel another larger study is required to provide further clarity in this matter.

**Level of evidence:**

Level II—comparative study.

## Introduction

Tibial fractures are common in ambulatory children [1]. Many different methods of fixation have been used, each with varying degrees of success [2–5].

External fixation has traditionally been favoured in fractures with soft-tissue problems, those with unstable fracture configurations and periarticular fractures [6–14]. Both monolateral and circular fixators can be used, although the circular fixator is a more stable construct biomechanically and may be more suitable for older children and fractures with an unstable configuration [13]. Ilizarov pioneered the use of the circular fixator for fracture treatment and deformity correction. The Taylor spatial frame (TSF) is a more recent circular fixator that uses a computer software programme for multiplanar correction of limb deformities. This has the advantage of being able to perform adjustments and fracture corrections postoperatively.

We have used both types of circular fixator for the treatment of tibial fractures. However, since 2005, the TSF has been the fixator of choice. In this study, we present our results relating to the treatment of tibial fractures with either an Ilizarov circular fixator or Taylor spatial frame.

## Methods

We have retrospectively reviewed all consecutive acute tibial fractures that were managed with circular fixators in our institute from 2000 to 2008. All patients had a minimum follow-up of 24 months. Ten patients were treated with the Ilizarov circular fixator (ICF), which was used until 2005. Fifteen further patients have since been managed with the TSF. Our indications for the treatment of a tibial fracture with a circular fixator were open fractures, fractures with unstable configurations, and fractures that displaced after initial treatment in a cast. Patients who developed compartment syndrome also had stabilisation with a circular fixator.

The patient details were obtained from the theatre database. Clinical and demographic data were acquired from the medical records. These included age, sex, mode of injury, other concomitant injuries, initial treatment, operative technique used, complications and duration of treatment. Radiographs were reviewed for assessment of initial injury, fracture reduction and alignment during the early postoperative period and to determine fracture healing.

**Fig. 1:**
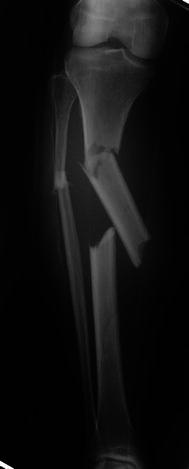
Preoperative radiograph of a segmental tibial fracture

**Fig. 2:**
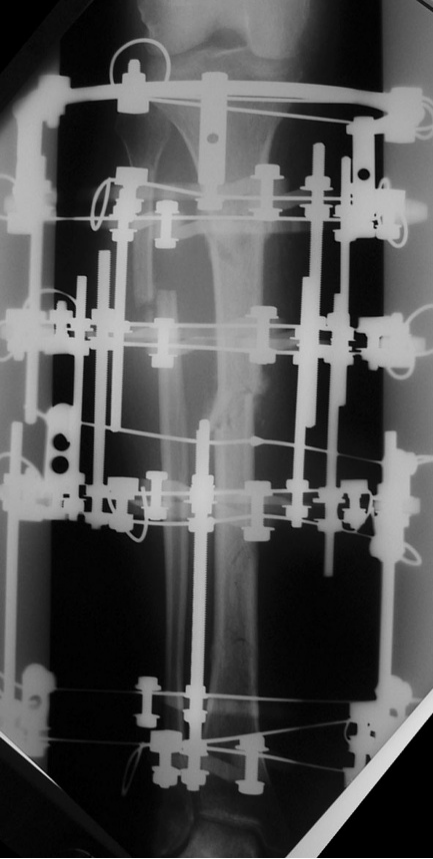
Taken during treatment once the Ilizarov frame had been attached

**Fig. 3:**
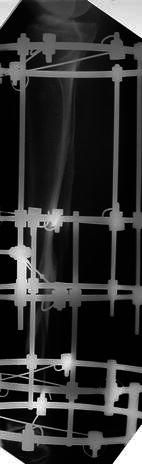
Showing sound union at the level of the original fracture

**Fig. 4:**
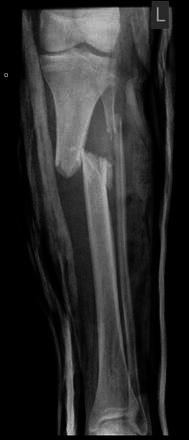
Preoperative radiograph of an unstable proximal 1/3 tibial fracture

**Fig. 5:**
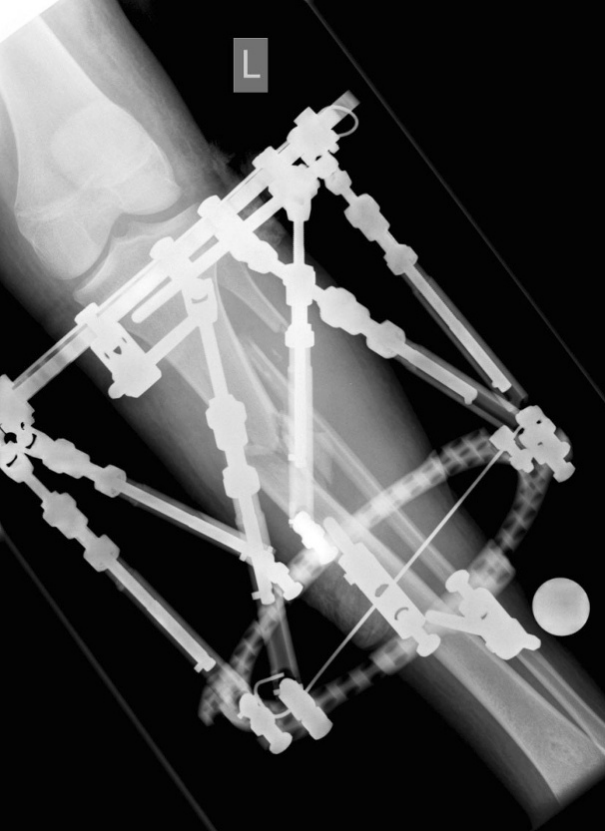
Immediate postoperative radiograph following stabilisation with TSF

**Fig. 6:**
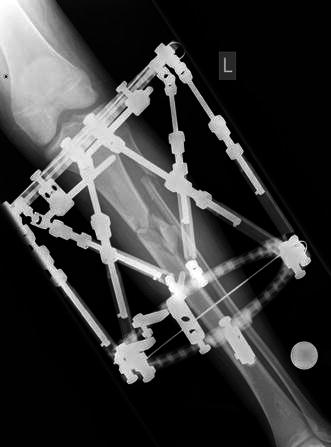
Following correction of the residual deformity with the TSF programme

The surgical technique was similar for both types of fixators. Surgery was performed in a laminar airflow theatre, which was our unit’s standard operating theatre used for such cases. All patients received antibiotic prophylaxis at induction of anaesthesia. Tourniquets were not used. Fluoroscopy was used for fracture reduction as well as for application of the fixator. The “ring first” technique was used, where the first reference wire was introduced into the segment for each ring, to which the ring was attached. For ICF, two rings were used per major segment, transfixed with olive wires and half pins (see Figs. 1, 2, 3). The wires were tensioned to 110 kg. Supplementary fixation techniques such as arched wires and push–pull wires were used in cases of oblique fracture configurations to minimise shearing at the fracture site. With the TSF, a two-ring configuration was used with two to three half pins and one or two wires per ring. We use 6-mm hydroxyapatite (HA)-coated half pins. The fractures were reduced to the best possible alignment and held in the desired position. Whenever possible, we prefer to use struts equipped with a fast closure mechanism as they conveniently lock fractures in a desired reduction. When further fracture reduction was required, it was performed postoperatively with the aid of internet-based software (Smith and Nephew Richards, Memphis, TN, USA) (see Figs. 4, 5, 6). The total residual programme was used in all cases. All patients in the TSF group had postoperative fracture corrections.

The patients were mobilised full weight bearing on the first postoperative day where possible, and were followed up after discharge at the outpatient clinic at weekly intervals until all of the corrections had been made. The decision to remove the frame was made if there was evidence of bridging callus at three cortices on the AP and lateral radiographs. The frames were removed in the operating theatre under general anaesthetic. A clinical assessment of the fracture site was made before complete removal. All patients were then protected in a below-knee walking cast for 4 weeks. If there was insufficient evidence of radiographic healing by 16 weeks, fracture union was considered to be delayed. The frame was dynamised for 4 weeks to stimulate healing. If, however, there was still insufficient fracture healing, we proceeded to iliac crest bone grafting of the fracture site and adjustment of the fixator at the same time if required. During follow-up, clinical and radiological assessments of alignment and length discrepancy were performed.

Statistical analysis was performed in SPSS 11.5. An independent sample *t*-test was used to determine the difference between means and the chi-squared goodness of fit test was used for proportions.

## Results

Twenty-five patients underwent circular frame fixation for their tibial fractures in our unit between the years 2000 to 2008. Among them, 10 patients were managed with the Ilizarov fixator and 15 patients were treated with the TSF. This was the primary treatment option for 8 patients in the Ilizarov group and 12 patients in TSF group. In the rest of the patients, conservative treatment was initially started, and circular fixators were applied due to loss of reduction in plaster. In all cases, this was performed within 2 weeks of injury. The mean age of the patients in both groups was 12.7 years. Half of the patients in the Ilizarov group were girls, whereas only 2 of the 15 in the TSF group were girls (Table 1).

**Table 1:** Summary of all patients with tibial fractures treated with circular fixators

	TSF	Ilizarov
No. of patients	15	10
Mean age (years)	12.7	12.7
Age range	7–15	9–15
M:F	13:2	5:5
Open fractures	5	2
RTA	8	3
Farmyard injury	0	1
Proximal metaphyseal–diaphyseal junction	2	1
Diaphyseal	4	4
Distal diaphyseal–metaphyseal junction	8	4
Segmental	1	1
Primary treatment option	12	8
Failure of conservative treatment	3	2
Mean time duration in frame (weeks)	14.8	12.7
Delayed union	2	0
Pin-site infection	9	7
Joint stiffness	1	1

Four patients in the ICF group sustained tibial fractures as a result of high-energy injuries, which were defined as occurring due to a high-speed road traffic accident (RTA), a fall from a significant height, and all were open fractures. Three of these were due to an RTA, of which two fractures were open. In the TSF group, eight fractures were the result of an RTA and five of these fractures were open. The segmental distribution of all the fractures as well as the number of open fractures is given in Table 1. All fractures in both groups went on to achieve complete union. The mean times for fracture healing were 12.7 weeks for the ICF (range 10–18 weeks) and 14.8 weeks (range 7–48 weeks) for the TSF group, respectively (*p* = 0.51). The average time for healing in patients where the circular fixator was applied as a secondary treatment option was 12.8 weeks in the TSF group and 13.1 weeks in the ICF group. For closed fractures, the mean healing time was 12.8 weeks (range 9–21 weeks), and for open fractures the mean healing time was 17 weeks (range 7–47 weeks).

Two patients in the TSF group (13 %) went on to develop a delayed union (healing time more than 16 weeks) and required further intervention with adjustment of the frame, supplemental fixation with HA-coated half pins, iliac crest bone grafting and fibular osteotomy. Healing times were 48 and 21 weeks, respectively (see Table 2). Both patients sustained the tibial fracture following an RTA and had sustained a head injury, with one of the patients sustaining a concurrent chest injury and forearm fracture. The fracture pattern in these cases was a short oblique fracture, and one of these patients had also sustained a Gustilo grade 1 open fracture that was treated with debridement and delayed closure. This patient also went on to develop malunion after the bone grafting procedure, and required an osteotomy to correct the deformity.

**Table 2:** Management of two cases of delayed union in the TSF group

	Time to healing (weeks)	Associated injuries	Fracture configuration/location	Open/closed	Length of stay (days)	Further treatment
Case 1	48	Severe head injury	Short oblique, segmental, mid-diaphysis	Open	60	Bone grafting, fibular osteotomy acute shortening of tibia + HA-coated pins
Case 2	21	Head injury Chest injury Fractured radius + ulna Degloving of foot	Short oblique, distal diaphyseal metaphyseal junction	Closed	31	Bone grafting, fibular osteotomy + HA-coated pins

A further patient in the TSF group did not show adequate signs of healing at 9 weeks post frame application and, at the discretion of the treating surgeon, underwent adjustment of the frame and iliac crest bone grafting at that stage. This fracture went on to heal by 15 weeks.

Three patients developed compartment syndrome following injury. In two of these patients, the primary treatment was closed reduction and cast stabilisation. Four-compartment fasciotomies were performed in all cases, and fractures were stabilised with a circular fixator. In all cases, delayed closure of the fasciotomy wound was performed.

Sixteen patients developed superficial pin-site infections; seven of these patients had ICF and nine had TSF (*p* = 0.78). There were no cases of deep infection. Only one patient required the removal of a loose wire because of recurrent infection. One of the patients who had delayed union (case 1) required intravenous antibiotics for pin-site infection. In all other cases, a course of oral antibiotics resolved the infection. Two patients (one in each group) developed stiffness of the ankle joint that required a course of physiotherapy. There were no refractures in either group.

At the final follow-up, none of the patients had a clinical leg length discrepancy of more than 1 cm. None of the patients had any rotational malalignment on clinical examination, and radiographic alignment was within 5° of normal on both AP and lateral radiographs.

## Discussion

We believe that this is the first study to compare the use of the ICF and TSF to treat tibial shaft fractures in children. The primary healing rate was 100 % for ICF and 87 % for TSF (a combined healing rate of 92 % for circular fixators). As this group included a significant number of fractures sustained due to high-energy injuries (40 % of ICF and 53 % TSF), the primary healing rate is impressive. In our study, particularly in the TSF group, there were only 2 girls and 13 boys, but we believe that this reflects the preponderance of boys who sustain significant tibial fractures. This is in concordance with previous studies of tibial fractures in children, with one study of TSF use in paediatric tibial fractures [10] reporting that all patients in their study were boys.

The use of external fixators to treat paediatric tibial fractures is well established [6–14]. Both monolateral and circular fixators have been used successfully. Monolateral fixators are considered to have an advantage in that they are technically easier to apply when compared to a circular fixator, but there have been concerns over an increased risk of loss of reduction with monolateral fixators, especially when used in older children [9–11]. Both Myers et al. [10] and Gordon et al. [9] found that the complications following treatment with monolateral fixators increased when they were used in children older than 12 years. When circular and monolateral fixators were compared for the treatment of paediatric tibial fractures, a significant number of patients with monolateral fixators had loss of reduction and developed malunion, in contrast to the patients treated with circular fixators [9]. When compared to monolateral fixators, circular fixators provided greater stability of the fracture, with less risk of loss of position of the fracture. Circular fixators have an advantage, given the configuration of tensioned wires and half pins, in that they minimise parasitic movement in angulation and rotation without eliminating elasticity (Catagni). There is also the potential to perform postoperative corrections to obtain acceptable reduction and alignment. The results of treatment of paediatric tibial fractures with circular fixators are reported to be excellent in the literature [6–11].

The use of TSF has been increasing in the UK over the past 10 years. It confers versatility in multiplanar correction of deformity with the help of an internet-software-based programme, which is both accurate and easy to use. However, reports in the literature on the use of TSF for paediatric tibial fractures are scarce [9–11]. Al Sayyad et al. [10], in their retrospective reviews of the results for nine patients (ten fractures) treated with TSF, found that all fractures healed at a mean of 18 weeks in the frame. One patient in their series required iliac crest bone grafting at 8 weeks to promote healing. This patient had sustained a comminuted fracture following a high-energy injury. Five of nine patients developed pin-site infection in their series [9]. Eidelman et al. [11] reported results of the treatment of paediatric tibial fractures with the TSF, and all of the fractures went on to unite at a mean of 11 weeks. They did not report any delayed union or nonunion in their series.

We had 15 patients in the TSF group, all of whom went on to complete union with a mean 14.8 weeks in the frame and an additional 4 weeks in a below-knee walking plaster. Two of the patients in this group developed delayed union and required additional procedures including iliac crest bone grafting to the fracture site. Both patients also required supplemental fixation with HA-coated half pins and adjustment of the frame. HA-coated half pins have been shown to increase the stability of external fracture fixation constructs [15]. Both patients had sustained a high-energy injury in a road traffic accident. One of the fractures was open (Gustilo 1), involving the diaphyseal region of the midshaft, and one was in the distal diaphyseal–metaphyseal junction.

In contrast, the mean time in frame for the ICF group was 12.7 weeks. None of the patients in this group developed delayed union. Patients had acute reduction of their fractures in theatre, and supplementary fixation techniques were used for unstable fracture patterns. We used olive wires, as described by Metcalfe et al. [16, 17]. Shear movements are known to cause delay in fracture healing. In an animal model, Augat et al. [18] showed a 36 % reduction in peripheral callus in the shear group as compared to the axial loading group. Alemdaroğlu et al. [19], in their retrospective review of the management of tibial fractures in adult patients, found a significantly shorter fracture consolidation time when supplemental fixation was used. They also reported significant differences in the healing times of patients who developed pin-site infections and those who did not, indicating that an unstable bone–fixator construct led to both events. In our study, only one of the patients with delayed union developed a pin-site infection that settled after a course of intravenous antibiotics. Our overall rates of pin-site infection of 60 % in the TSF group and 70 % in the ICF group may seem high, but they are consistent with pin-site infection rates reported in the literature [10]. Except for one patient, all of these infections settled after a course of oral antibiotics and, as mentioned by Paley [20], we believe that such pin-site infections represent a “problem” rather than a true complication.

There are several techniques that may be used to improve stability in an unstable fracture configuration such as an oblique fracture. Metcalfe et al. found that push–pull wires and arched wires were most effective at reducing shear. They also found that a steerage pin was more effective than a transverse pin in these circumstances. These techniques are easier to use with the Ilizarov frame, and this may be a reason why there was no delayed union in this group.

In conclusion, we feel that both the fixators were excellent modes of fixation in this small group of patients with unstable fracture configurations. With TSF, there are the advantages of easy application and a versatile indirect fracture reduction method. In our opinion, for most paediatric tibial fractures, a standard technique of a two rings–two pins construct may work well, but we feel that techniques that would improve the stiffness of the frame should be used in all these cases, particularly when dealing with high-energy unstable fracture patterns. The use of 2–3 olive wires per ring along with 2–3 half pins in the maximum possible spread and divergence would allow adequate stability and stiffness. Spanning ankle and knee joints for proximal and distal fractures would also improve stability, as would the use of a smaller ring size distally. Our study was too small to look into these variables. We feel that further research, both biomechanical and clinical with the TSF, is needed to fully understand the various properties of the fixator in order to maximise its use.
